# Fatigue Life Assessment of Notched PLA Manufactured Using FDM 3D-Printing Technique

**DOI:** 10.3390/polym18010001

**Published:** 2025-12-19

**Authors:** Mahsima Seifollahi, Mohammad Zaman Kabir

**Affiliations:** Department of Civil and Environmental Engineering, Amirkabir University of Technology (Tehran Polytechnic), Tehran 15875-4413, Iran; mahsima.seifollahi@aut.ac.ir

**Keywords:** fatigue, FDM, build orientation, notch, failure mechanism

## Abstract

Fused Deposition Modeling (FDM) is an extensively employed additive manufacturing method for producing precise and complicated polymer models, with its industrial applications expanding under various loading conditions. A review of existing research highlights the insufficient investigation of the influence of geometric discontinuities in additively manufactured polylactic acid (PLA) members under fatigue loads. This study aims to analyze the combined effects of build orientation and geometric discontinuities on the static and fatigue performance and damage evolution of 3D-printed PLA. To achieve improved fabrication quality and minimize process-induced defects, the quasi-static tensile tests were conducted on specimens printed in on-edge orientation with a concentric infill pattern and the flat direction with a rectilinear infill pattern. The test results have shown that on-edge-printed objects have reduced micro-voids and improved layer bonding, resulting in a 19% increase in tensile strength compared to the flat-printed specimens. Consequently, this configuration was adopted for three specimen types, e.g., smooth, semi-circular edge-notched, and central-holed, tested under axial fatigue with a 0.05 load ratio. Fatigue test findings indicate that the stress concentration is more pronounced around central holes than near edge notches, leading to shorter fatigue life. This phenomenon is consistent with its effects under static tensile loading. Furthermore, using Digital Image Correlation (DIC) technique, damage initiation, progression, and failure mechanisms were analyzed in detail. According to fractographic analysis, the micro-voids in the 3D-printed specimens serve as potential regions for the initiation of multiple fatigue cracks. Additionally, the inherent internal defects can interact with geometric discontinuities, thereby weakening the fatigue performance.

## 1. Introduction

Additive Manufacturing (AM) technique offers advanced capabilities for constructing precise physical models with numerous geometries at relatively low costs using digital models. With AM, it is possible to produce complex structures that would be extremely challenging or impossible to fabricate through conventional manufacturing approaches. Additionally, AM supports using various materials, including metals, alloys, plastics, composites, and concrete [1,2,3,4]. These advantages expand the potential applications of AM across a wide range of industrial and technological fields. This cutting-edge technology provides mechanical engineering, automotive, aerospace, and construction industries with the fabrication of complicated and lightweight components, as well as rapid prototypes. In addition, it significantly contributes to reducing material waste and environmental impact [1,5,6]. Moreover, AM has introduced significant advantages in biomedical sectors, including medical devices, dentistry, and implant fabrication [7,8,9].

Fused Deposition Modeling (FDM), an extrusion-based 3D-printing technique, is a widely adopted and cost-effective manufacturing method that enables easy, fast, safe, and efficient operation. This method, mainly using thermoplastic filaments, employs a layer-by-layer approach to manufacture components directly from computer-aided design (CAD) models. A computer-controlled nozzle deposits melt thermoplastic material onto the heated build plate following the G-code commands, where it solidifies quickly and bonds with the adjacent material. After printing each layer, the build plate moves down to print the next layer [1,10,11,12,13]. Polylactic acid (PLA) is an eco-friendly biodegradable thermoplastic that shows comparatively high mechanical performance, low printing temperature, and superior processability, thereby becoming one of the most commonly used materials in FDM. This material has been increasingly used in industry for biodegradable textile components, medical prostheses and implants, rapid prototyping and design verification in mechanical engineering, as well as various applications across agriculture and food industries [14,15,16].

Industrial components are commonly subjected to various cyclic loading conditions, including mechanical and thermal fatigue [17]. Nowadays, with the improvements in polymer materials, their applications in the aerospace and automotive industries under mechanical cyclic loading conditions are expanding [18]. However, some internal defects due to the manufacturing process in 3D-printed polymers can serve as potential fatigue crack initiation regions. Hence, FDM components tend to exhibit weaker performance under fatigue loading than those manufactured through conventional methods, which noticeably restricts their industrial applications [18,19,20,21,22]. Gaps and micro-voids between printed filaments formed by the layer-by-layer nature of the FDM process are considered internal imperfections and can act as stress concentrators [23,24,25,26]. Apart from those, inter- and intra-layer bonding qualities are the main cause of the issues in FDM-processed parts [27,28,29].

Previous studies highlight that the optimization of printing parameters—such as melting temperatures [30,31,32], layer dimensions [21,33,34], infill pattern and density [33,35,36], build orientation, and raster direction [37,38,39]—can effectively minimize process-induced defects and improve the mechanical characteristics, structural integrity, and fatigue performance of FDM components made from different thermoplastic materials [18,24,26]. In light of these studies, it is evident that among these parameters, raster direction and build orientation, as the main sources of anisotropy in FDM parts, are particularly critical for achieving optimal printing configuration and fatigue performance. Thus, this topic has received increasing attention in the literature. Concerning raster direction, notable experimental studies have examined the fracture behavior of 3D-printed PLA manufactured with different raster angles under cyclic loading [40,41]. Their findings collectively demonstrated that the raster angle influenced the size and distribution of micro-voids in the cross-section of the specimen. Furthermore, the filament alignment relative to the loading direction served as a critical factor for the pathways of load transfer and crack propagation. Consequently, components with a raster direction of 0° enabled a more uniform load distribution across their filaments, resulting in higher uniaxial fatigue lifespan.

Following raster direction, considerable research has also focused on the role of build orientation in the fatigue durability of FDM components. Fischer and Schöppner [42] analyzed the uniaxial fatigue performance of additively manufactured Ultem 9085 in different build orientations (*XY* (Flat), *YZ* (on-edge), and *XZ* (upright)). The results indicated that at higher loads, specimens printed in the *YZ* orientation exhibited the highest life, followed by *XY* and *XZ*. The following research investigated the flexural fatigue behavior of FDM-processed polycarbonate (PC) [43]. Analysis of the fracture mechanism indicated that the localized maximum stress at interlayer interfaces in *XZ*-oriented specimens led to the poorest fatigue resistance. In line with the observed advantages of the *YZ* orientation over *XY*, Terekhina et al. [44] specified that reduced porosity in FDM-PA6 fabricated in the *YZ* orientation led to enhanced flexural fatigue life. Subsequently, extending these investigations, Hassanifard and Hashemi [45] employed an analytical strain-based approach—a method rarely applied in FDM fatigue studies—to more quantitatively assess the influence of build orientation on fatigue life. Taking into account all these studies, it is evident that build orientation influences fatigue resistance not only by controlling load distribution but also by interacting with microstructural defects, such as voids and interlayer bonding. In spite of these insights, a comprehensive understanding of how build orientation influences the microstructure, defect formation, and fatigue fracture mechanisms of 3D-printed PLA remains lacking.

Apart from inherent defects, which can be reduced by optimizing printing parameters, functionally required discontinuities, such as holes, notches, and grooves, are commonly present in the geometry of industrial components. Such geometric defects result in stress concentrations, which can significantly degrade the structural integrity of components and their mechanical performance. Hence, it is of great significance to assess the effect of such features on the service life of 3D-printed parts under cyclic loading, thereby enabling the safe design of industrial components. In this context, numerous works have investigated the effects of geometric discontinuities and their interaction with the internal gaps and voids on the strength of FDM components under static loads [46,47,48]. Nevertheless, this aspect has been less extensively studied in the context of fatigue performance. Several studies have examined the fatigue behavior of edge notches with different sharpness levels and central notches with varying aspect ratios in PLA and reinforced PLA printed in the *XY* orientation. The results indicate that the severity of these discontinuities depends on both their sharpness and the printed raster direction. For instance, in PLA printed with a 90° raster angle, the gaps between the filaments made the central notches more pronounced than those in other specimens [49,50,51,52]. Therefore, the evaluation of geometric discontinuities must be carried out with consideration of their interaction with internal defects.

In line with the literature, existing evidence indicates that the fatigue behavior of FDM components is governed not only by material properties but also by the interaction of process-induced defects, functionally required discontinuities, and printing parameters such as build orientation. Nevertheless, their combined influence on the static and fatigue behavior of 3D-printed PLA—particularly at the microscale—has yet to be comprehensively investigated. Moreover, the macrostructural mechanisms that control fatigue damage initiation, growth, and final fracture have received limited attention, hence creating a notable gap in understanding the damage process in PLA components. Thus, the present research aims to address these gaps by experimentally evaluating the impact of *YZ* (on-edge) orientation on the tensile properties of FDM-processed PLA. In addition to the existing knowledge gap on the fatigue behavior of this material, PLA was chosen for its current wide usage in functional prototypes and lightweight components, its ready availability, and dimensional stability. The main contribution of this work is the experimental investigation of geometric discontinuities on the tensile and fatigue performance of *YZ*-oriented PLA with a concentric infill pattern, offering a deeper understanding of their role in damage evolution. To do so, Scanning Electron Microscopy (SEM) imaging is used to investigate fracture mechanisms and the interaction between interior gaps and geometric imperfections. Additionally, Digital Image Correlation (DIC) was employed—a technique rarely used in previous works—to identify fatigue crack initiation, its growth rates, and failure in notched PLA components. The outcomes of this research are anticipated to be of use for reliable design by enhancing the knowledge of fatigue behavior in additively manufactured PLA, a widely used plastic material.

## 2. Materials and Methods

### 2.1. Material and Specimen Fabrication

**Figure 1:**
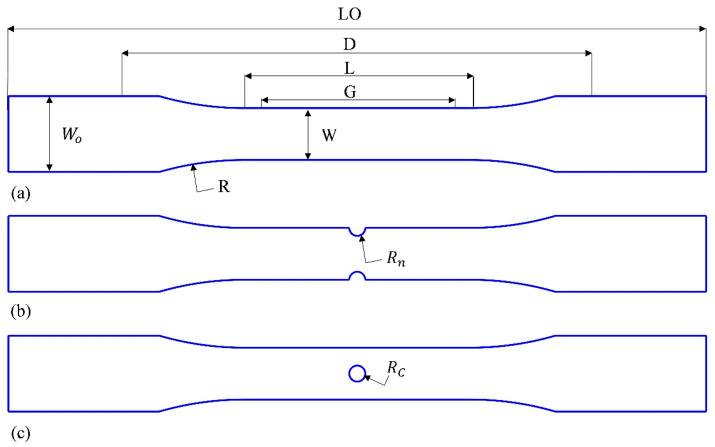
(**a**) The geometry parameters of a Type I tensile specimen according to the ASTM D638 standards, (**b**) edge-notched specimens, and (**c**) central-holed specimens (LO: length overall, D: distance between grips, L: length of narrow section, G: gage length, W: width of narrow section, WO: width overall, R: radius of fillet).

In this study, all tensile and fatigue test specimens were produced using FDM on the HERMES 3D-printer (Isfahan, Iran). Commercial white PLA filament from Yousu (Guangzhou, China), with a diameter of 1.75 mm, a density of 1.24 g/cm^3^, and composed of more than 98% pure polylactide resin, was utilized as the raw material. To ensure material consistency and improve the reliability of the experimental results, a single PLA filament spool was used throughout the study. ASTM D638-10 [53], standard test method for tensile properties of plastics, is recommended by ASTM D7791-12 [54], standard test method for uniaxial fatigue properties of plastics, as the reference for selecting the specimen configuration for uniaxial fatigue testing. Consequently, the Type I geometry specified in ASTM D638-10 [53] was adopted for all specimens, with the geometry shown in Figure 1a and corresponding dimensions listed in Table 1. For a larger grip area, the overall length of the specimens was raised by 10 mm in addition.

To investigate the influence of build orientation on the tensile strength of PLA manufactured via FDM, the test specimens were fabricated in both *XY* (Flat) and *YZ* (on-edge) orientations with a 0° raster direction. The remaining printing parameters were kept constant for all 3D-printed specimens. The nozzle and build plate temperatures were adjusted to 215 °C and 55 °C, respectively, with a nozzle diameter of 0.4 mm and a layer height of 0.2 mm. Moreover, the infill density was set to 100% to minimize gaps and voids within the specimens. Considering the limitations of the machine and ensuring suitable quality, the printing speed of 3200 mm/min has been chosen.

**Figure 2:**
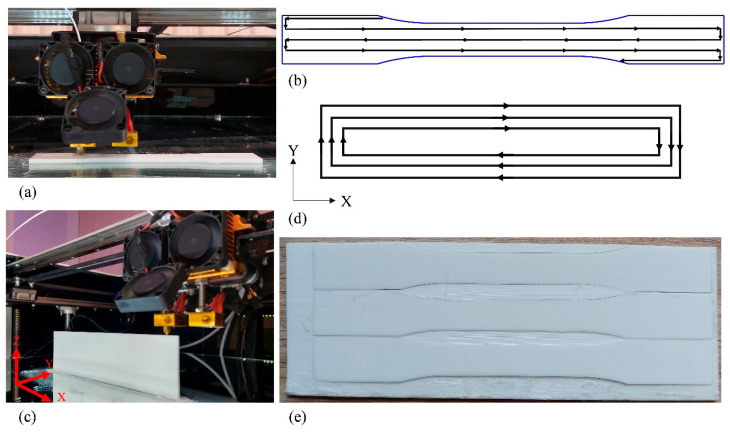
*XY* specimens: (**a**) printing process and (**b**) rectilinear infill pattern; *YZ* plates: (**c**) printing process, (**d**) concentric infill pattern, and (**e**) laser-cut plate.

The specimens built in the *XY* direction were printed individually with the rectilinear infill pattern (Figure 2a,b). The printing infill pattern may induce non-uniformity and a limited molten zone in printed objects due to the initiation and cessation of the filament printing process [55]. In this study, *YZ* specimens were manufactured by carrying out a recently proposed printing method that overcomes these issues by providing a uniform microstructure [56]. The concentric infill pattern was considered for printing rectangular plates with dimensions of 67 mm by 195 mm in the *YZ* orientation, Figure 2c,d. Afterward, plates were cut by laser along the filament direction to provide the final *YZ* specimens illustrated in Figure 2e. To investigate the fatigue behavior of *YZ* specimens containing initial geometric discontinuities, two different notch shapes, both of which had a radius of 2 mm, were examined: a central hole and a semi-circular edge notch, as illustrated in Figure 1b,c. The notches were introduced into the *YZ* specimens through laser cutting. Following the manufacturing of the specimens, the width and thickness, as well as the diameters of the holes and edge-notches, were measured with a digital caliper. The actual thickness and width of the specimens deviated by up to 0.1 mm from the nominal values due to unavoidable variations in the manufacturing process, while the notch sizes were exact. These small deviations were within acceptable limits and were taken into account in the study (see Table 2).

### 2.2. Quasi-Static Tensile and Fatigue Test

**Figure 3:**
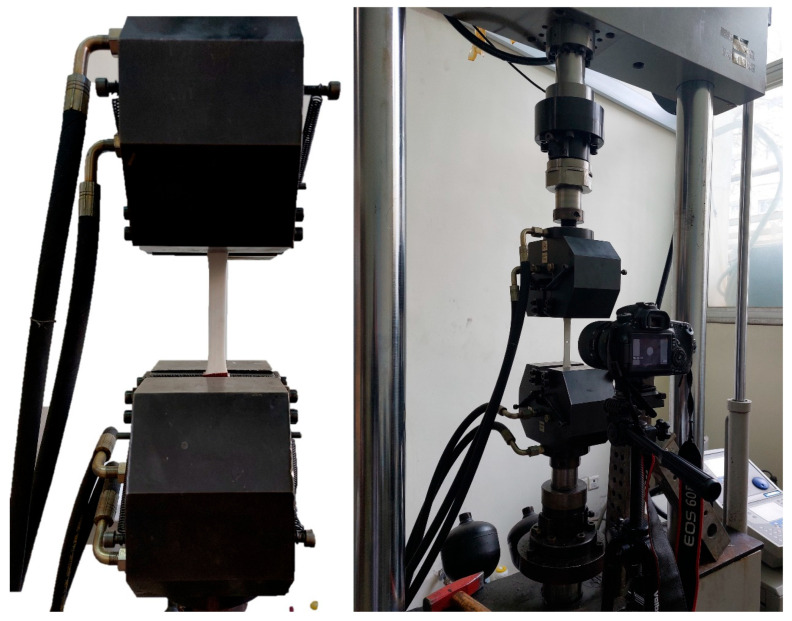
Fatigue and quasi-static tensile test set-up.

To evaluate the influence of build orientation and notch shapes on the tensile mechanical properties and fatigue performance of 3D-printed PLA specimens, it was necessary to experimentally determine their tensile and fatigue characteristics. To ensure comparability with prior studies, all tests were conducted in compliance with the relevant standards. The tensile tests were performed in accordance with ASTM D638-10 [53], under the displacement-controlled and quasi-static loading condition with a speed of 1 mm/min. Moreover, a Dartec servo-hydraulic tensile/fatigue testing machine (Stourbridge, UK) at the Aerospace Fatigue Laboratory was used for this purpose. Each test was repeated three times per specimen type to ensure consistency in the obtained results. Moreover, the fatigue tests were conducted on the same machine following ASTM D7791-12 [54] to examine the performance of notched PLA under fatigue loading. To do so, the smooth and defective specimens, fabricated in the *YZ* orientation, were subjected to sinusoidal axial fatigue loading at a frequency of 5 Hz and a load ratio σminσmax of 0.05 until a complete fracture occurred. Although ASTM D7791-12 [54] recommends at least three repetitions per stress level, practical constraints—namely, the time-consuming nature of the fatigue tests and specimen preparation—limited the number of fatigue tests performed. With this in mind, multiple repetitions were carried out at all four stress levels; however, only the most representative results were reported. The applied loads and stress levels are summarized in Table 3, while the fatigue test setup is depicted in Figure 3.

### 2.3. Fatigue Fracture Process

#### 2.3.1. Fatigue Crack Propagation Monitoring

DIC is a non-contact optical method to measure full-field displacement and strain on the surface of deforming materials. This effective technique was used to analyse local strain and detect the initiation and propagation of fatigue cracks in the notched specimens. In addition, crack tip positions, as well as crack size and growth rate, were identified and measured using the data obtained from DIC [57,58,59]. Images for DIC analysis were captured by securely positioning a commercially available Canon 60-D camera (Tokyo, Japan) with an effective resolution of 18 megapixels in front of the test setup. The focus was calibrated to ensure clarity in the notched area of the specimen, which represents the potential final fracture zone. To achieve DIC results, it is essential to generate a recognizable speckle pattern on the specimen for tracking through image processing. Accordingly, the surface of the specimens was first coated with glossy white spray paint to cover the relatively rough surface caused by the layer-by-layer manufacturing process. Afterward, a matte black spray powder-sized particles was applied to the specimen surface to create a proper, randomly distributed spot design. The recorded images were analyzed using the open-source Ncorr software version 1.14 in MATLAB R2022a. The notched area of the specimen was determined as the Region of Interest (ROI) to analyze the strain and crack growth process. The subset size and strain radius are the main parameters used for calculations. The subset refers to the region of the image tracked by the algorithm across frames, and the strain radius is defined as the radius of the circle used to select a group of points for plane fitting. Both parameters were selected to be as small as possible to avoid introducing noise into the displacement and strain data, respectively. Ultimately, the deformations and strains were utilized as output results to assess fatigue crack initiation and the growth process.

#### 2.3.2. Fractographic Examination Using SEM

Examining SEM images of fractured surfaces provides insights into the behavior of the material and the failure mechanisms of 3D-printed PLA specimens subjected to quasi-static tensile and fatigue tensile loading conditions. Additionally, these images help in analyzing the influence of notches on the failure process. Hence, the fractographic analysis was performed using an AIS 2300C SEM (Seron Technologies, Uiwang-si, Republic of Korea) operated at an accelerating voltage of 20 kV. SEM images were captured at various levels of magnification, and each image included corresponding scale bars to ensure reproducibility of the observations.

## 3. Results and Discussion

### 3.1. Quasi-Static Tensile Test

#### 3.1.1. *XY* and Smooth *YZ* Specimens

**Figure 4:**
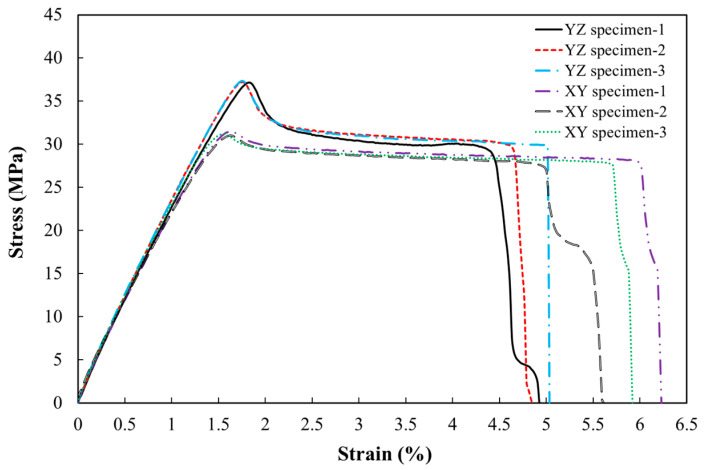
Stress–strain curve of *XY* and *YZ* specimens.

**Figure 5:**
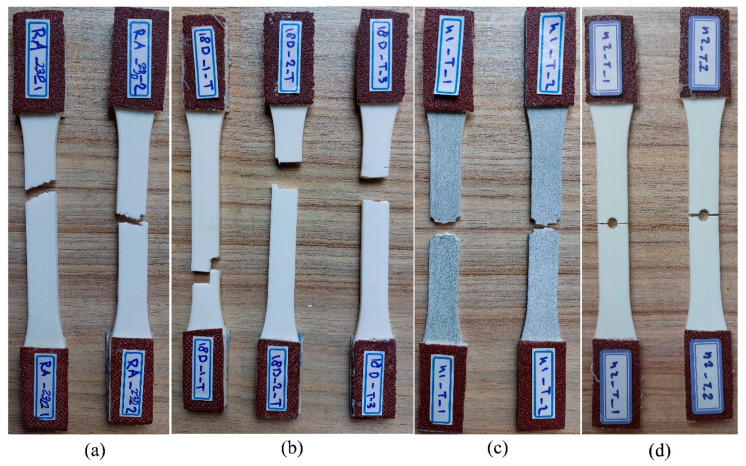
The broken tensile specimens: (**a**) *XY*, (**b**) *YZ*, (**c**) edge-notched, and (**d**) holed specimens.

*XY* and *YZ* specimens were subjected to the static tensile tests in order to examine the effect of build orientation on the static tensile properties of additively manufactured PLA. Figure 4 illustrates the stress–strain relationship for both *XY* and smooth *YZ* specimens. The key test results, including Young’s modulus, ultimate tensile stress, and the corresponding elongation at the final stage, for each specimen, are presented in Table 4. In addition, for the purpose of statistical analysis and assessment of variability within each group, the 95% confidence intervals (CIs) were calculated to indicate the range in which the true mean is expected to lie. Overall, the results showed a relatively similar response regarding Young’s modulus and the ultimate tensile strength among the three repeated specimens. However, a slight difference was noted in the obtained measurements due to inevitable deviations in the printing parameters. Furthermore, to compare the tensile performance of *XY* and *YZ* specimens, *p*-values for the mentioned parameters were obtained using Welch’s t-test, due to the limited number of tests and unequal standard deviations (SDs) between groups. Based on Figure 5a,b, which illustrates the broken *XY* and *YZ* specimens, it was evident that the fracture was occurred within the gauge length of all dog-bone shaped specimens.

The stress–strain behavior of smooth *YZ* tensile specimens, as shown in Figure 4, has indicated an average ultimate tensile strength of 37.234 MPa, corresponding to a strain of 1.83%. The curve demonstrated an almost linear response up to approximately 70% of the ultimate tensile strength, a region where Young’s modulus can be determined. Beyond this point, phenomena such as yield softening, plastic deformation, and localized necking in some filaments led to a sharp non-linear drop in the stress–strain curve [56]. The observed drop in the curve indicates a two-step softening phenomenon, attributed to the premature failure of outer filaments and the subsequent redistribution of stress to inner filaments in early plastic deformation [56]. As the loading progressed, the material degradation occurred, resulting in a significant loss of load-carrying capacity and an accelerated strain rate until the final rupture at approximately 4.937% tensile strain. The stress–strain curve of the *XY* specimen (Figure 4) demonstrated a similar linear trend up to 80% of the peak load. However, beyond the peak, the curve has exhibited a gradual, non-linear drop. The mean final failure strain of 5.9%, which represented an increase of approximately 17% compared to the *YZ* specimen, highlighted this matter. Additionally, the average ultimate tensile strength of the *XY* specimen was approximately 31.2 MPa, about 19% lower than that of the *YZ* counterpart. Statistical analysis, as presented in Table 4, revealed that the *p*-values for ultimate tensile and elongation at break were almost 0 and 0.00217, respectively—both well below 0.05—indicating a statistically considerable influence of the build orientation on the strength and ductility of 3D-printed PLA. By contrast, according to the *p*-values of 0.65 for Young’s modulus, well above 0.05, the build orientation had little to no effect on its stiffness. Moreover, such mechanical differences may, in part, be attributed to microstructural variations arising from the printing orientation, which were examined through SEM imaging.

**Figure 6:**
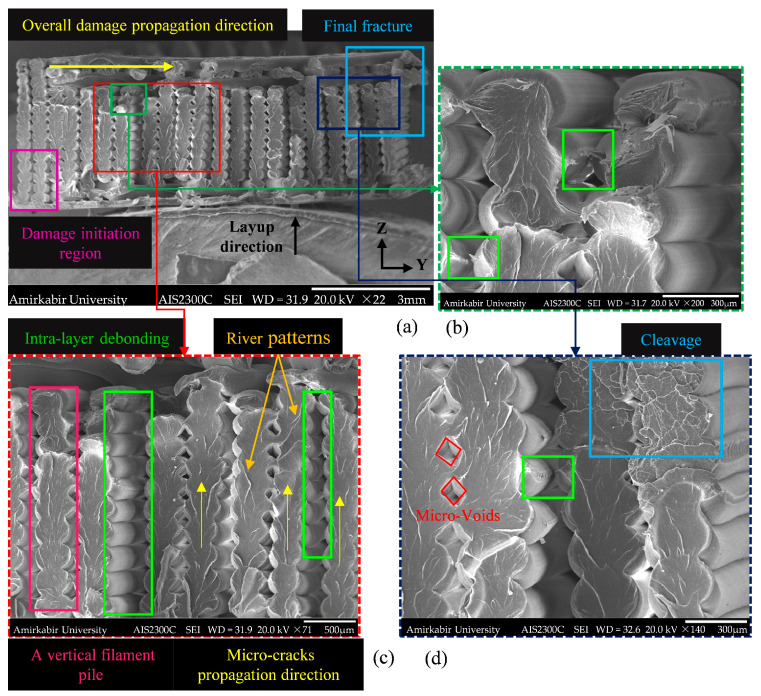
The fractured surface of the tensile *XY* specimen (RA-23-D1) captured via SEM at various magnifications: (**a**) ×22, total cross-section; (**b**) ×200, intra-layer debonding; (**c**) ×71, characteristics of damage mechanisms; (**d**) ×140, micro-voids and cleavage. All explanation text is colored to match the corresponding boxes and arrows in each image.

The fracture surface of the *XY* specimen, which was printed with a rectilinear infill pattern, is demonstrated using the SEM technique in Figure 6a. According to the separated filaments marked in Figure 6b,d, the inter-layer bonding in the *XY* specimens was relatively weak. By increasing the tensile load, the poor bonding resulted in intra-layer debonding as the primary failure mode. Also, the smooth lateral surfaces of some filaments in the *XY* specimen (Figure 6c) may indicate that the debonding among certain filaments was referred to an initial process-induced imperfection.

**Figure 7:**
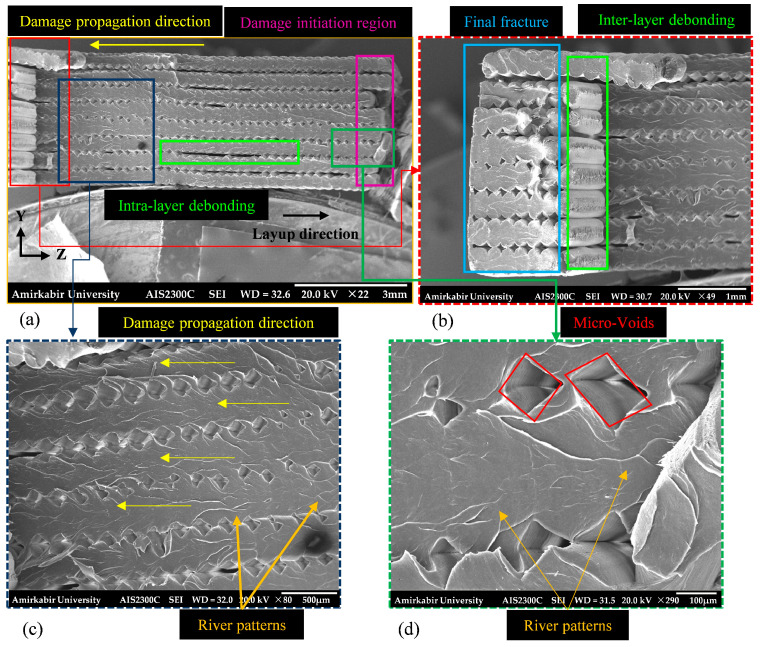
The fractured surface of the tensile *YZ* specimen (18-2-T) captured via SEM at various magnifications: (**a**) ×22, total cross-section; (**b**) ×49, final fracture zone; (**c**) ×80, damage propagation zone; (**d**) ×290, micro-voids and river patterns. All explanation text is colored to match the corresponding boxes and arrows in each image.

In contrast, as shown in Figure 7, the *YZ* printed specimens with a concentric infill pattern exhibited strong intra- and inter-layer cohesion, with adjacent filaments within the same layer and successive layers fully fused. Despite this, a few minor debondings remained visible. The designated region on the lateral surface with considerable roughness in the lateral surface of filaments, see Figure 7b, specifies filament separation. Other than increased inter-layer fusion, considering the fracture surfaces of both types of specimens, the *YZ* specimens contained fewer micro-voids compared to the *XY* specimens. Therefore, the *YZ* printing orientation combined with the concentric infill pattern fully utilizes the load-bearing capacity of the filaments, resulting in stronger and more structurally integrated specimens.

Figure 6d demonstrates the damaged surface of the *XY* specimen with cleavage marks, representing a low-energy, brittle fracture. The river patterns depicted in Figure 6c denote cleavage fracture surface features. The river pattern consisted of a network of cleavage steps formed by connecting cleavage planes throughout the fracture development.

These patterns could be utilized to determine the direction of the damage, as the convergence of the river pattern features follows the crack growth path [60]. The damage initiation zone is shown in Figure 6a, with the final fracture zone located on the opposite side. The overall damage propagation occurred along the longitudinal axis of the specimen. However, based on the observed river pattern, it was evident that as the load increased, the local damage in the form of micro-cracks progressed through adjacent filaments printed in successive layers, aligned with the transverse axis of the specimen Figure 6c. The damage development can be described as follows: micro-cracks originated from the bottom side of a filament and were transmitted along the layup direction. The damage process of vertical filament piles was formed consecutively, and one by one, the filament piles failed until the final one yielded to failure. The discussed mechanism causes stress concentration at the bonding interface between two adjacent vertical filament piles until debonding.

The fracture surface of the *YZ* specimen revealed cleavage and river patterns, as illustrated in Figure 7c,d. Analysis of the river pattern on the fracture surface indicated that damage progressed in the layup direction, corresponding to the longitudinal axis of the specimen. Due to the printing orientation, both the overall and local damage propagation directions were aligned, minimizing significant intra- and inter-layer debonding.

#### 3.1.2. Edge-Notched and Central-Holed *YZ* Specimens

**Figure 8:**
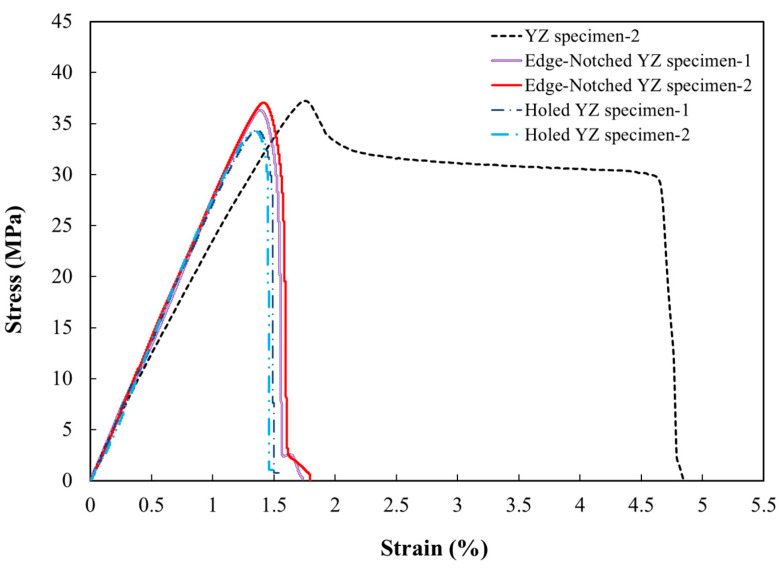
Stress–strain curve of edge-notched and holed specimens.

To assess the impact of edge notches and central holes on the tensile strength of FDM-processed PLA, the stress–strain curve of notched and smooth specimens is compared in Figure 8. According to the results summarised in Table 5, the specimen containing a central hole with a radius of 2 mm shows the lowest ultimate tensile strength with 34 MPa, reflecting a reduction of approximately 8% in comparison to the reference smooth specimen. As depicted in Figure 8, the stress–strain curves for notched specimens show a predominantly linear trend up to the ultimate tensile strength, beyond which a sharp failure drop was observed, indicative of brittle behaviour. This is completely in contrast to that of the smooth specimens, which perform with considerable plastic strain. The experimental results have also indicated that the edge-notched specimens failed at a tensile strength of around 36.6 MPa, which is 1.5% less than the reference one. These non-pronounced effects of edge notches and central holes on the ultimate tensile strength of defective specimens compared to reference ones may refer to the nature of the PLA additive manufacturing method in which the internal voids were formed, diminishing the overall impact of the edge notch on the measured mechanical properties. In addition, Figure 5c shows that fracture in the edge-notched tensile specimens occurred within the notched region.

**Figure 9:**
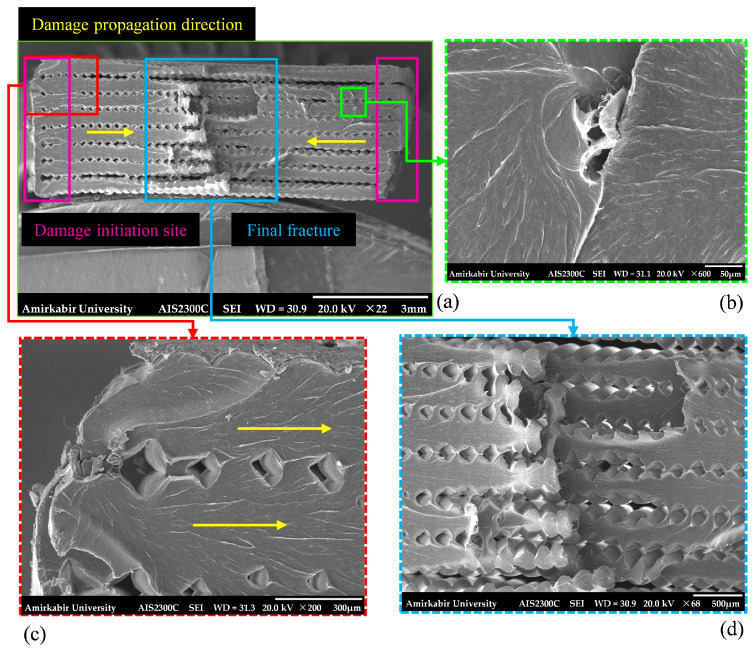
The fractured surface of tensile edge-notched specimens captured via SEM at various magnifications: (**a**) ×22, total cross-section; (**b**) ×600, a process-induced defect; (**c**) ×200, damage initiation zone; (**d**) ×68, final fracture zone.

Figure 9 shows the SEM pictures of the damaged surfaces of the edge-notched specimens. Figure 9a,c displays that the cracks originated at the notch roots and propagated towards the center of the section as the applied load was increased with gradual filament breakage. When the cracks were merged, rapid failure occurred owing to the brittle fracture in the middle of the gauge length, representing an acceptable failure mode. According to Figure 9d, at the point of crack combination and the stage of the final failure, some filaments were pulled out, and the debonding failure mode was evident, without significant plastic deformation. Furthermore, as shown in Figure 9b, the river pattern indicates that micro-cracks have originated from the void between printed filaments, suggesting that process-induced defects act as potential regions for damage initiation.

**Figure 10:**
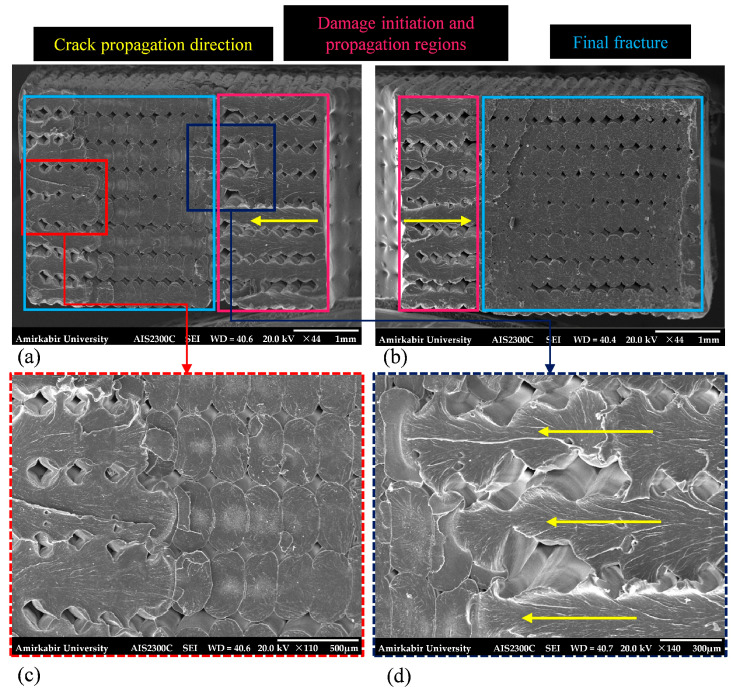
The fractured surface of tensile-holed specimens captured via SEM at various magnifications: (**a**) ×44, the left half of the sample; (**b**) ×44, the right half of the sample; (**c**) ×110, final fracture zone; (**d**) ×140, crack propagation zone.

According to the experimental practice, a similar phenomenon occurred in the specimens featuring central holes, where these holes served as critical sites for crack initiation. Based on Figure 10a,b, the cracks were initiated from both sides of the hole and propagated through the damaged section until the final fracture took place. The SEM images, see Figure 10a,c,d, display that the initiation and propagation region containing river patterns and a rapid propagation to the final fractured area were separated by an obvious border. Also, the final fracture surface was wider and smoother than the initiation area, indicating brittle fracture behavior, consistent with the characteristics observed in the stress–strain curve.

Given that the edge notch and the central open hole had the same radius of 2 mm, the remaining load-bearing areas in both cases were equal. Therefore, this comparison highlights the influence of the shape of geometric discontinuity on the material mechanical performance. When assessing the behavior of edge-notched versus central-holed specimens, it became apparent that the presence of a central hole introduced a more critical mechanical condition, as it induced a greater reduction in load-bearing capacity and ultimate tensile strength. This observation specified that the stress concentration around the central hole was more severe than around the edge notch, contributing to the higher potential for failure in the central-holed specimens. Conversely, the presence of a notch had a relatively minor effect on the ultimate tensile strength. The explanation for this phenomenon could be pronounced as follows: Considering that the notch was positioned at the edge of the specimen, whereas the hole was centrally located, the material around the hole experienced greater confinement, restricting stress release. In contrast, the deformation of the edge notch, accompanied by localized strain, facilitated partial relief of the concentrated stress in that region.

### 3.2. Fatigue Results

#### 3.2.1. Smooth *YZ* Specimens

**Figure 11:**
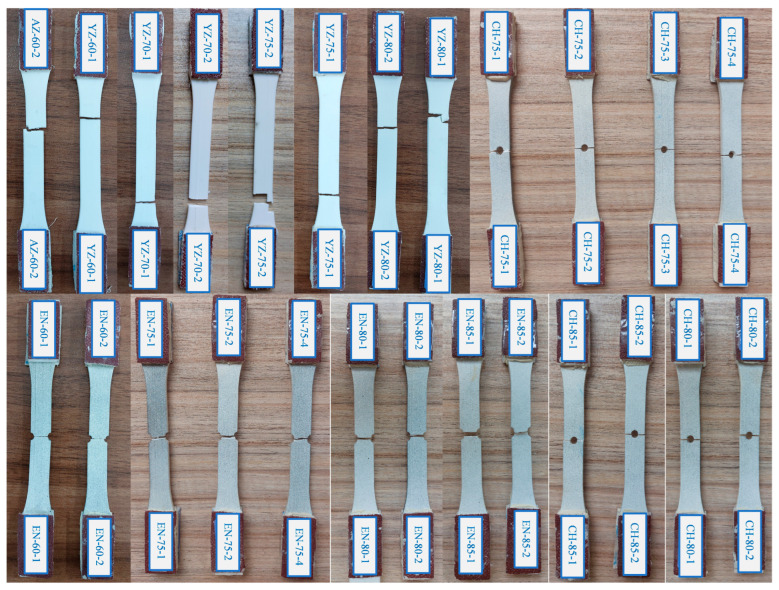
The broken smooth, edge-notched, and central-holed specimens.

Three types of specimens—unnotched, edge-notched, and central-hole—were tested under fatigue loading to assess the impact of notches on the fatigue performance of PLA built in the *YZ* orientation. Figure 11 displays the fractured specimens subjected to the fatigue loading as described in Section 2.2. The smooth *YZ* specimens were broken within the gage length, while the defective specimens experienced failure at the notched area, as predicted.

**Figure 12:**
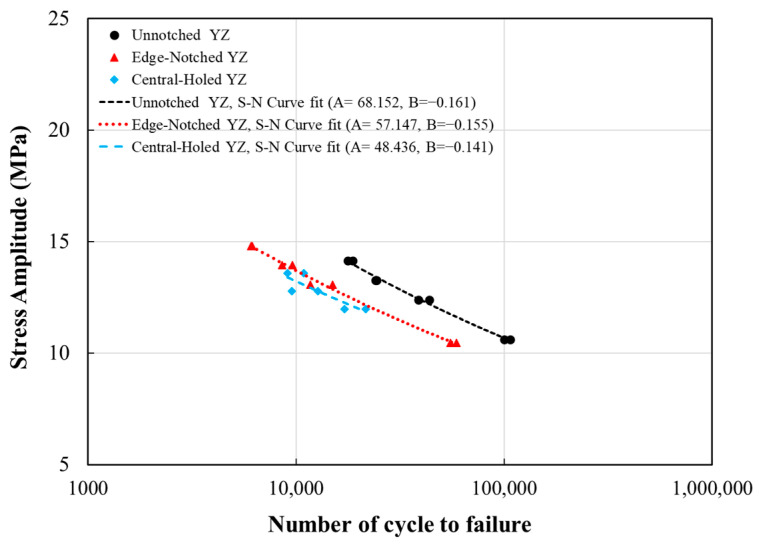
S-N curve for smooth, edge-notched, and holed specimens.

The results of the axial fatigue tests conducted on specimens are presented in (S-N) diagrams on a semilogarithmic scale in Figure 12. The fatigue results are summarized in Table 6, showing the fatigue life of each specimen under individual stress levels. Despite performing several repetitions at each stress level, unreliable data—such as premature failures and results exhibiting abnormal deviation—were excluded, and consequently, two representative fatigue lives were reported for each stress level. According to ASTM E739-10 [61], the minimum number of test specimens required for research and development testing of components ranges from 6 to 12. Furthermore, the minimum acceptable percent replication, which can be obtained using Equation (1), for this purpose is 33–50%. As shown in Table 6, both the minimum number of data points and the 50% replication for all three testing groups fall within the acceptable range, thereby ensuring the reliability of the reported data and the resulting S–N curves.

(1) $$Replication\%=100.(1-\frac{Total\;number\;of\;different\;stress\;levels}{total\;number\;of\;specimen\;tested}).$$

The S-N curves were built using a power law formula as follows:

(2) $$\sigma_{amp}=A{{\;.\;N}_{f}}^{B}.$$

where $\sigma_{amp}$ is stress amplitude, N_f_ is fatigue life, and *A* and *B* are fatigue coefficients which were calculated based on curve fitting of experiments. The evaluated fatigue coefficients for each specimen type, and R^2^ values—representing the accuracy of the curve fit to the experimental data—are summarized in Table 7. Concerning the reported R^2^ values, those relevant to the smooth and edge-notched specimens are sufficiently close to one, while the value of 0.736 for the central-holed specimens is relatively low, showing the scatter in the fatigue data. This scatter tends to be caused by errors, such as inaccuracies in the experimental setup and measurements. Moreover, in 3D-printed notched specimens, manufacturing variability—particularly internal defects and layer bonding—and stress concentrations around the notch are the main sources of inconsistencies.

According to the observed results in the S-N curve for FDM-processed PLA fabricated in the *YZ* orientation, the fatigue life was deteriorated as the applied stress level increased, as expected. Specifically, a 16% increase in the maximum applied stress, from 22.34 MPa to 26.06 MPa, led to a 60% reduction in fatigue life. Similarly, a 7% rise in maximum stress, from 26.06 MPa to 27.92 MPa, caused the cyclic lifespan to reduce by 40%. However, at the higher stress levels, a 7% increase, from 27.92 MPa to 29.78 MPa, resulted in a 25% reduction in the number of cycles to failure.

To investigate the impact of build orientation on the fatigue behavior of 3D-printed PLA, the fatigue test results from this study were compared to the S-N curves for PLA specimens that were fabricated in the *XY* orientation, as reported in the literature [41,50,52,62]. The fatigue test data for the *YZ* specimens were obtained at a load ratio of 0.05, while the *XY* specimen data outlined in [52] were collected at a load ratio of 0.1. Hence, a mean-stress correction was required to ensure full comparability between the data sets. For this purpose, the Smith–Watson–Topper (SWT) approach was used to standardize all fatigue results to a fully reversed loading condition (R = −1). Fundamentally, the SWT method is an analytical approach that combines the maximum stress and the strain amplitude within a single stress–strain function, enabling the prediction of fatigue life of structures. In this framework, the influence of mean stress on fatigue behavior is incorporated across a wide range of load ratios, under the assumption that fatigue damage is primarily governed by the tensile portion of the loading cycle. In addition, the main advantage of this approach is its independence from specific material constants, as it relies solely on data obtained from a small number of simple fatigue specimens. Moreover, the SWT method is applicable to a broad spectrum of materials, including those that exhibit only small plastic strains. However, in materials with complex behavior and significant plasticity, this method may provide a slightly non-conservative prediction. Apart from that, in more complex structures, the SWT approach is limited to crack initiation and early propagation, without capturing full fracture behavior at the critical location. Due to the fact that the 3D-printed PLA specimens in this study exhibited an almost brittle behavior with limited plasticity under the applied loading conditions, the use of this approach is justified. Hence, the SWT formulation presented in Equation (3) is employed to determine the completely reversed stress amplitude ($\sigma_{amp,R=-1}$) for the fatigue tests performed at a load ratio of 0.05 under various maximum stress levels [63,64].

(3) $$\sigma_{amp,R=-1}=\sigma_{max}\left[\sqrt{\frac{1-R}{2}}\right].$$

**Figure 13:**
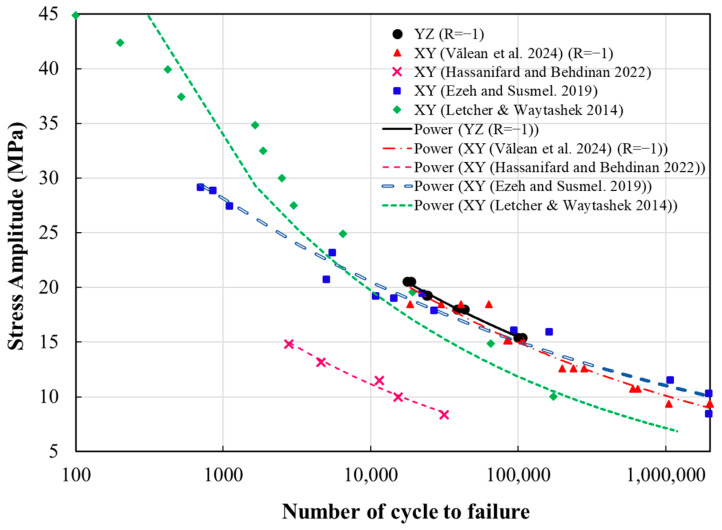
S-N curve for *XY* and *YZ* specimens with a load ratio of −1 [41,50,52,62].

Additionally, S-N curves for each data set were fitted using a power-law model, see Figure 13. Accordingly, the S-N curves for the PLA specimens built in the *XY* orientation and 0° raster direction, as documented by Ezeh and Susmel [41], Hassanifard and Behdinan [50], and Todd Letcher [62], are located below and close to the curve of *YZ* specimens, demonstrating their slightly enhanced fatigue resistance. Likewise, the S-N curve of the specimens printed using + 45/−45 layup in the *XY* orientation, as reported in [52], was close to that of *YZ* specimens, and the *YZ* specimens exhibited a marginally longer fatigue life than the *XY* components at the same stress level.

#### 3.2.2. Edge-Notched and Central-Holed YZ Specimens

The geometry of discontinuity on the fatigue performance of FDM-processed PLA is considered a critical aspect in fatigue analysis. A comparison study of the fatigue performance results from experiments on edge-notched, central-holed, and smooth specimens has been carried out. It was evident that there was a significant reduction in the fatigue resistance of those 3D-printed PLA specimens possessing geometrical defects. This reduction was observed as a downward shift in the S-N curve for smooth specimens (see Figure 12). The S-N curve for central-holed specimens was positioned below that of edge-notched specimens. Based on the derived S-N relationships, at a stress amplitude of 12 MPa, a smooth specimen can endure approximately 49,000 load cycles, while central-hole and edge-notch specimens can resist around 24,000 and 20,000 cycles, respectively. In other words, the specimen with an edge notch results in a 50% reduction in the cycle number at the stage of failure, whereas a central hole makes an even more reduction in fatigue life, approximately 60%.

The fatigue notch factor (K_f_) is defined as for a given number of cycle, the ratio of stress amplitude of smooth specimen over the stress amplitude of defected specimen. This may apply to standardized fatigue data for a fully reversed loading condition via the SWT approach. For a semi-circular edge notch and a central hole with a radius of 2 mm, the K_f_ was obtained as 1.124 and 1.155, respectively, which are close to each other. The main reason for the K_f_ values staying close to 1 may be associated with the gaps and voids within the specimen, due to the manufacturing process, which has dominated the induced defects, e.g., edge notch and circular hole. In other words, the inherent defects have facilitated the initiation of fatigue cracks and fatigue life. Apart from that, the K_f_ value at high stress amplitude, which causes intermediate and short fatigue lives, remains relatively small and close to 1 [63].

### 3.3. Analysis of Fatigue Fracture Mechanism

#### 3.3.1. Fractography

**Figure 14:**
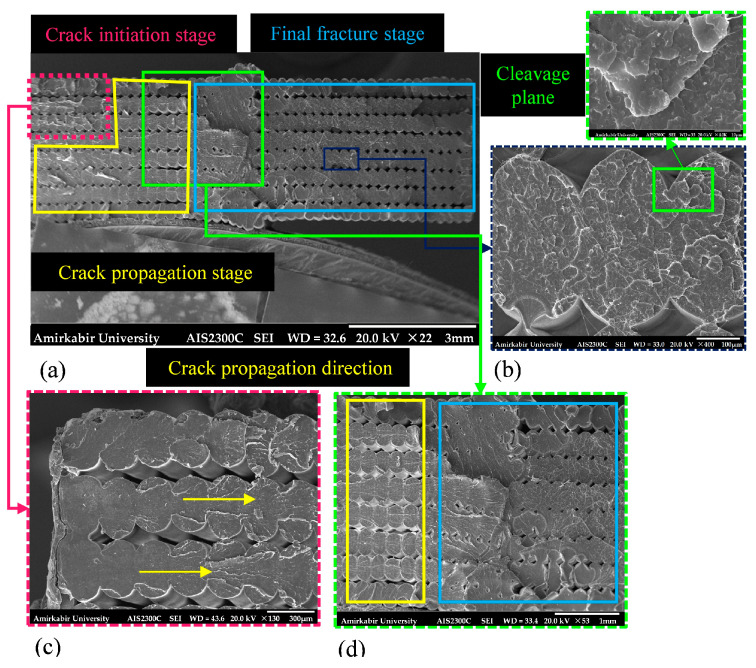
The broken surface of the fatigue smooth *YZ* specimen (*YZ*-70-2) at a stress amplitude of 12.38 MPa with different scales: (**a**) ×22, total cross-section; (**b**) ×400, final fracture zone including cleavage planes; (**c**) ×130, damage initiation zone; (**d**) ×53, damage propagation and final fracture zone. All explanation text is colored to match the corresponding boxes and arrows in each image.

**Figure 15:**
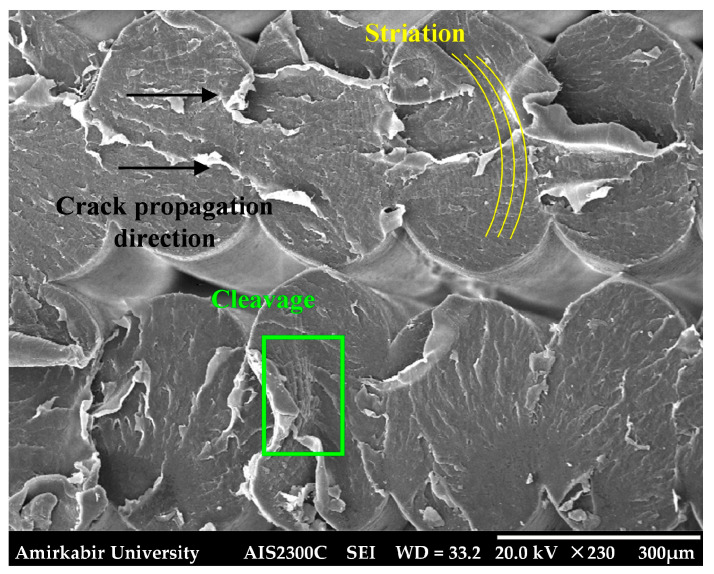
Magnified view of filaments failed in stage II of fatigue fracture in fatigue smooth *YZ* specimen.

Fractography, as a reliable technique for analyzing fatigue phenomena, was employed in this study to present a precise understanding of the underlying fracture mechanisms. In this section, the results obtained from the SEM analysis are discussed. The fracture surface of the smooth *YZ* specimen, which failed under fatigue loading at a stress amplitude of 12.38 MPa, is depicted in Figure 14. The surface distinctly revealed the three stages of fatigue fracture: fatigue crack initiation, crack propagation, and eventual catastrophic failure. A fatigue crack nucleated on one side of the specimen, as designated by a dashed line in Figure 14a,c, and was propagated by enduring the fatigue loading. Stage II fatigue was characterized by the presence of striations, which serve as a visual record of the position of the fatigue crack tip as it propagates through the material [60]. These crack-arrest marks, illustrated in Figure 15, radiate away from the crack initiation region, indicating the direction of crack growth. The striations are generally oriented perpendicular to the principal crack propagation direction.

**Figure 16:**
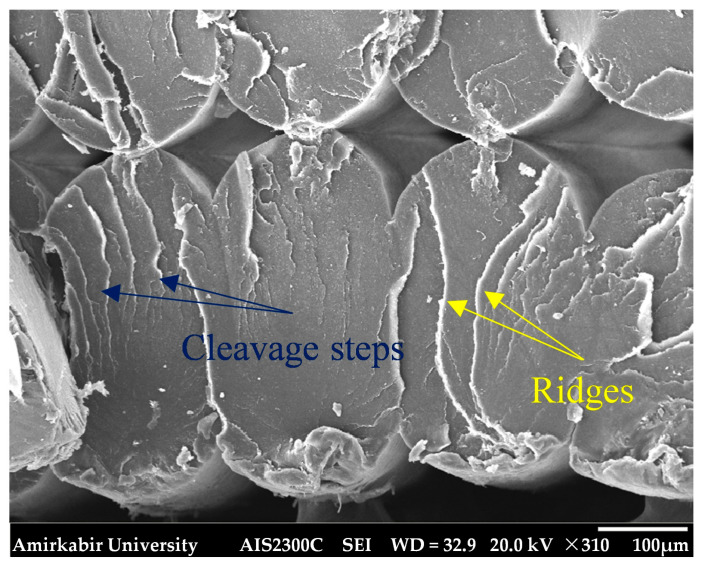
Magnified view of filaments failed in stage III of fatigue fracture in fatigue smooth YZ specimen.

Moreover, Stage III of fatigue fracture, see Figure 14b,d, demonstrating the unstable crack growth to the final fracture zone, exhibited a relatively smooth surface in contrast to the rough and irregular facets characteristic of the crack propagation region observed in Stage II. Cleavage features, indicative of a low-energy fracture, were visible on the fracture surface and are highlighted in Figure 16. These features suggest that the filaments were broken in a brittle mode. In addition, some filaments have contained ridges, indicating rapid advance of the crack and sudden fracture [60].

**Figure 17:**
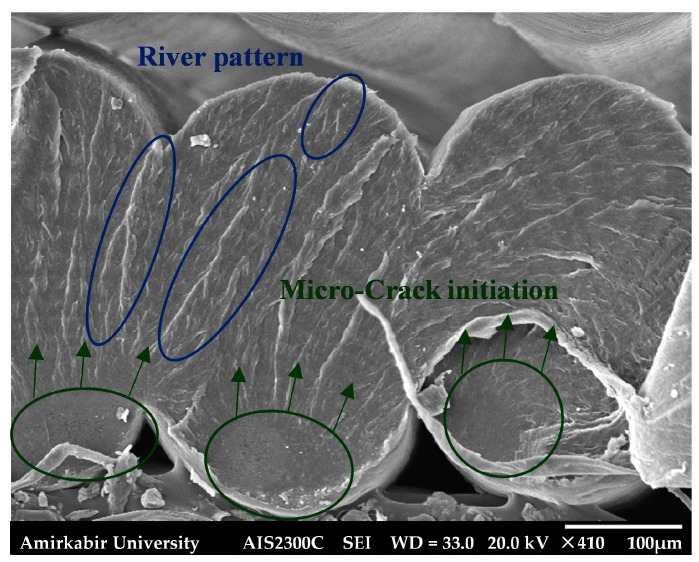
The final layers of the fracture surface of the fatigue smooth *YZ* specimen.

In the final layers of the fracture surface, see Figure 17, debonding between some filaments was observed. Micro-voids between the printed filaments acted as internal defects, causing local stress concentration points. These defects resulted in multiple fatigue cracks and localized micro-cracks nucleated on each filament. The different directions of local micro-crack growth could be distinguished from the river patterns visible on the filaments.

**Figure 18:**
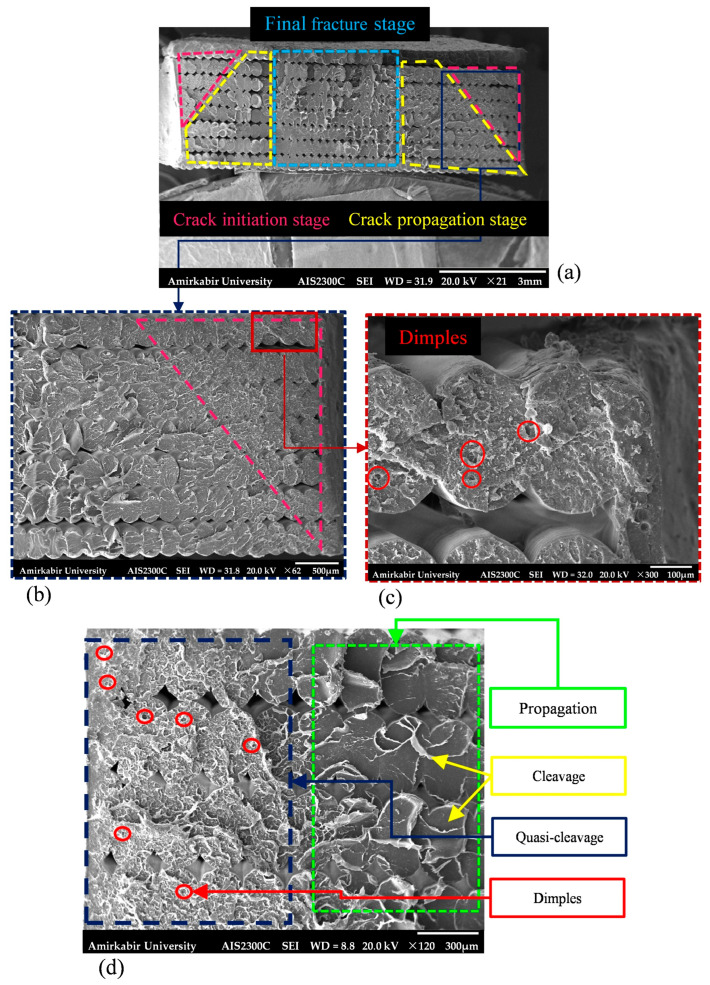
The broken surface of fatigue edge-notched specimen at (**a**–**c**) 75% and (**d**) 80% of its ultimate strength captured via SEM at various magnifications.

Figure 18 shows the fractured surface of edge-notched specimens subjected to fatigue loading at 75% and 80% of their ultimate strength. In the specimen tested at 75% of its ultimate strength, crack initiation was rooted at both notches, characterized by a highly rough surface. Additionally, the crack initiation regions, as illustrated in Figure 18c,d, in both specimens exhibited features of quasi-cleavage, displaying a combination of dimples and cleavage. A similar mechanism was reported in the study by Azadi et al. [65]. Quasi-cleavage refers to a fracture mechanism characterized by isolated, localized features on the fracture surface that combine the qualities of both cleavage and plastic deformation. It originates at the central cleavage facets, which gradually blend into regions of dimple rupture as the crack propagates, with the cleavage steps changing into tear ridges [60]. A transition from a uniaxial to a biaxial and ultimately to a triaxial state of stress reduces material deformation. The restriction on plastic deformation, e.g., a triaxial stress state which forms near notch roots, can provide the quasi-cleavage fracture [60,66]. As shown in Figure 18d, this mechanism occurred adjacent to the notch root during the crack initiation stage, and the fracture mode was changed to cleavage throughout crack propagation.

**Figure 19:**
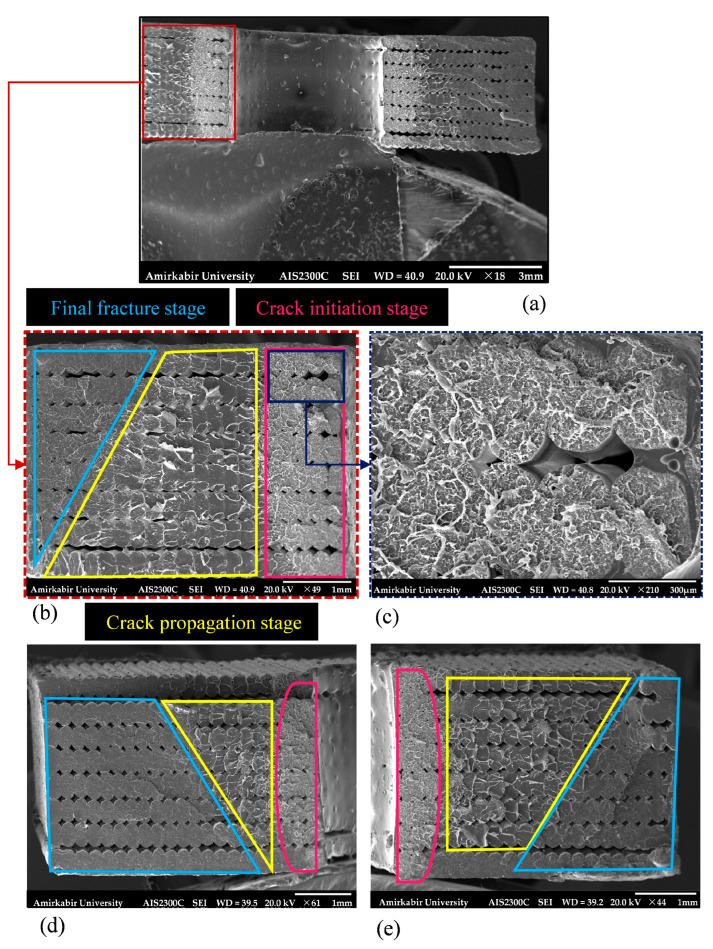
The broken surface of the fatigue central-holed specimen at (**a**–**c**) 75% and (**d**,**e**) 85% of their ultimate strength captured via SEM at various magnifications.

Referring to Figure 19b,c, a similar behavior is observed in central-holed specimens on both sides of the hole. As shown in Figure 19b, the fatigue cracks were developed near the central hole and propagated through three distinct stages. The size of these stages varies depending on the applied stress levels. In specimens tested at 75% of their ultimate strength, the crack initiation and growth area was larger, while the final fracture area was smaller compared to those tested at 85% (Figure 19d,e).

#### 3.3.2. Fatigue Damage Evolution

**Figure 20:**
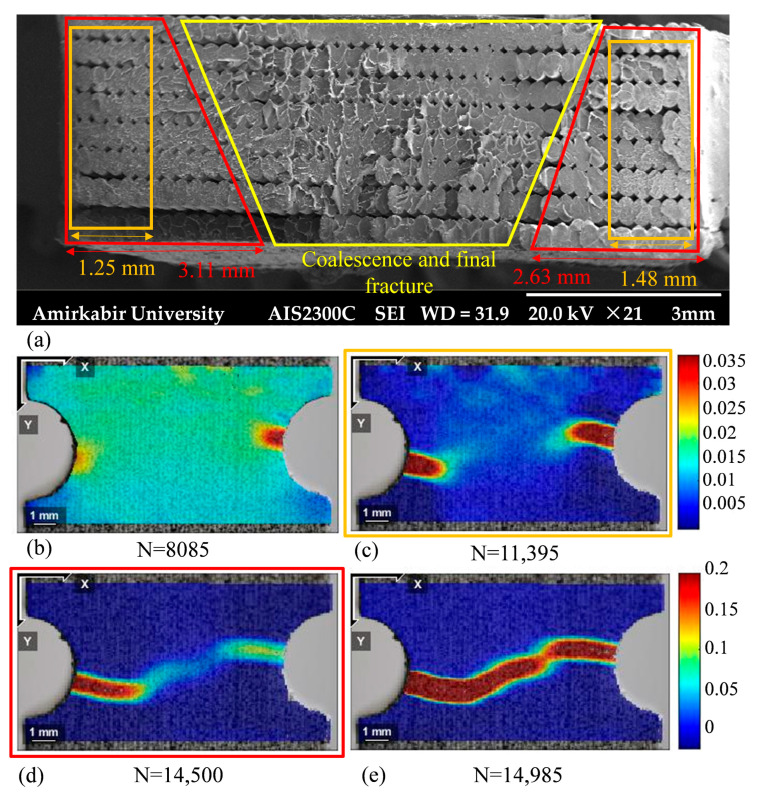
Fatigue damage progression of edge-notched specimen at 75% of its ultimate strength: (**a**) SEM images of the fractured surface, (**b**–**e**) strain distribution in the y-axis at different cycles.

**Figure 21:**
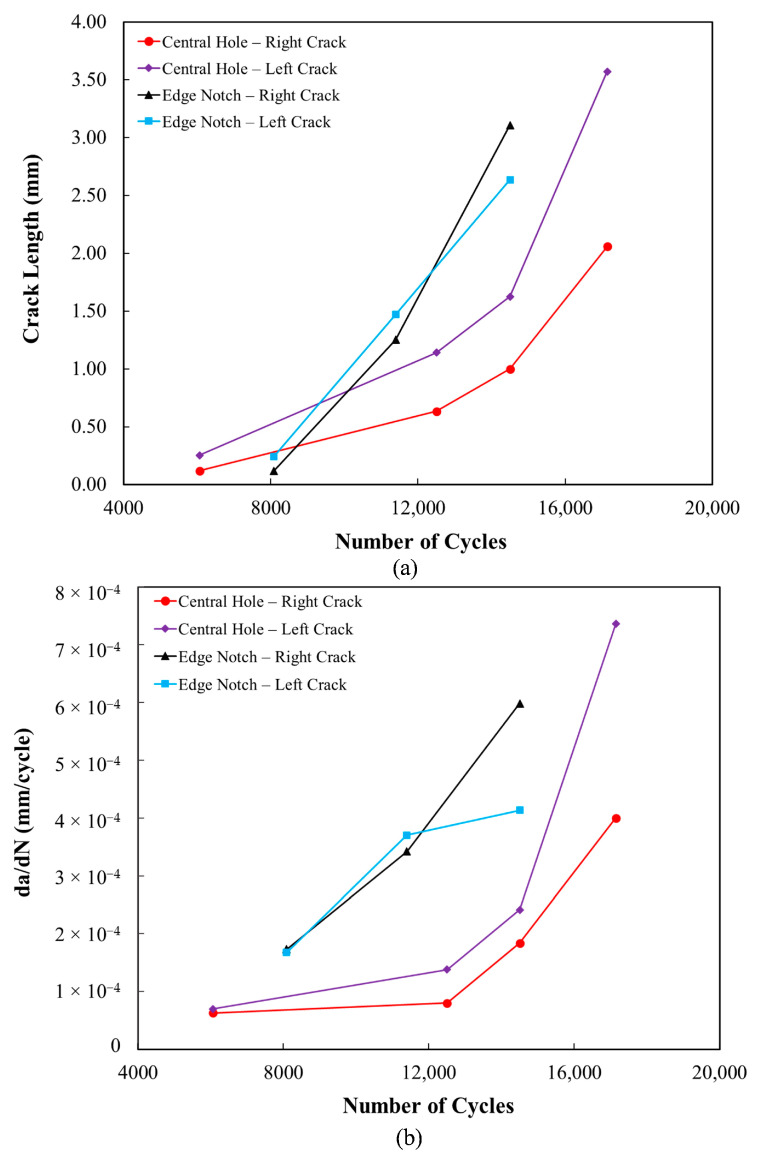
(**a**) Crack length against cycles number. (**b**) Crack growth rate against cycles number.

The fatigue crack initiation and growth process during fatigue loading in the vicinity of the edge notch and central hole was monitored via DIC. The strain distribution in the edge-notched specimen at a maximum nominal stress of 27.52 MPa at different cycles is illustrated in Figure 20. Additionally, Figure 21 represents the changes in crack length and growth rate (da/dN) versus cycle number. Given the definition of crack growth rate as the change in crack length per cycle, and considering a simplified approximation, this parameter was obtained using the following equation [63]:

(4) $$\frac{da_{i}}{dN_{i}}\approx \frac{∆a_{i}}{∆N_{i}}=\frac{a_{i}-a_{i-1}}{N_{i}-N_{i-1}}.$$

where $a_{i}$ and $N_{i}$ represent the ith crack length and number of cycles, respectively. In the fracture surface of the specimen, see Figure 20a, the fatigue damage process corresponding to each DIC image is highlighted using color codes that match the frame colors of the respective DIC images. According to Figure 20b, the strain concentration near the notches indicates that the crack nucleation and micro-crack growth phase continued up to approximately 8085 cycles, with a growth rate of around 1.74 × 10^−4^ mm/cycle. This period represented 53% of the total fatigue life, during which the crack size reached approximately 0.244 mm. Throughout this phase, the notch-emanating crack growth rate is influenced by the longitudinal concentrated stress distribution ahead of the notch tip. As the crack propagates away from the notch, the crack tip moves out of the region of notch-induced stress rise. By doing so, the crack was propagated at a rate equivalent to that of a single crack with a total length of the notch and the crack size [67].

Furthermore, according to the strain distribution of the edge-notched specimen, see Figure 20d, unstable shear flow occurs in the bond between the two cracks at 14,500 cycles, with the crack sizes measuring 3.11 mm and 2.63 mm, respectively, see the confined region with red line border in Figure 20a. The phenomenon, known as the crack coalescence, redirects the crack growth paths toward each other [68]. Ultimately, the coalescence process lasted fewer than 500 cycles in an unstable manner before the final fracture failure, while the crack growth rate was increased from 5.98 × 10^−4^ to 0.01 mm/cycle (see Figure 20e). Additionally, the final fracture zone, marked in yellow in Figure 20a, exhibits a rough, non-coplanar surface, consistent with the DIC image at the failure cycle.

**Figure 22:**
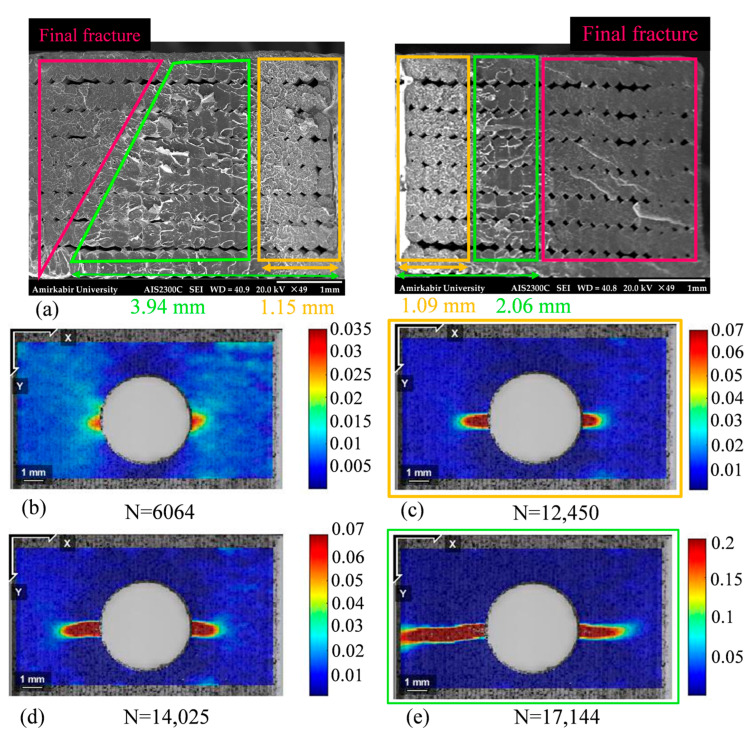
Fatigue damage progression of central-holed specimen at 75% of its ultimate strength: (**a**) SEM images of the fractured surface, (**b**–**e**) Strain distribution in the y-axis at different cycles.

Similarly, the initiation and the development of the fatigue crack failure in the central-holed specimen were observed at the maximum nominal stress of 25.23 MPa, as explained in Figure 22. By referring to the strain distribution of the central-holed specimen shown in Figure 22b, the fatigue damage initiates from both sides of the hole at around 6064 cycles. As fatigue load progresses, the cracks continue to grow at a relatively slow rate, around 1.09 × 10^−4^ mm/cycle. At this stage, the cracks reached the lengths of 1.15 mm and 1.09 mm by 12,450 cycles, which represents 72% of the total fatigue life. Subsequently, the crack—marked in green in Figure 22a—propagates at an increasing rate, accelerating to 7.36 × 10^−4^ mm/cycle until final failure occurs at 17,164 cycles, which is highlighted in pink in Figure 22a.

## 4. Conclusions

In this work, the mechanical performance of PLA fabricated via FDM was explored under static and fatigue loading. As far as industrial applications are concerned, geometrical discontinuities are unavoidable in many functional components—such as mechanical joints and fixtures with screw holes. Therefore, both smooth specimens as reference samples and those containing edge notches or central holes were investigated to evaluate the influence of such features on the fatigue life of 3D-printed PLA. In addition, Smooth tensile test specimens were manufactured in both the *XY* and *YZ* orientations to assess the effect of build orientation on tensile properties. Moreover, fatigue damage evolution and fracture mechanisms were further analyzed using DIC and SEM techniques. The key results are as follows.

The average tensile strength of the *YZ* specimens with a concentric infill pattern was 19% higher than that of the *XY* specimens with a rectilinear infill pattern, highlighting the notable impact of build orientation. Fractographic analysis revealed that *YZ* orientation combined with enhanced inter- and intra-layer bonding and reduced process-induced micro-voids. Quasi-static tensile tests on semi-circular edge-notched and central-holed PLA specimens showed that the geometric discontinuities in 3D-printed PLA caused a significantly brittle fracture, exhibiting no noticeable deformation. Moreover, the presence of a central hole decreased the ultimate tensile strength by 8%, while an edge notch resulted in only a 1.5% reduction.

A comparison of the S-N curves from fatigue tests indicated that geometric discontinuities, acting as a stress concentrator, substantially degraded the fatigue resistance of FDM-processed PLA. In this context, central holes were more deleterious than edge notches, leading to a greater reduction in fatigue lifespan. Notably, the influence of geometric discontinuities on fatigue notch factor values remained relatively minor, as process-induced voids served as inherent imperfections, facilitating the evolution of fatigue damage.

The fatigue damage analysis of smooth and defective specimens revealed that the internal defects, such as micro-voids within the 3D-printed part, contributed to the initiation of multiple fatigue cracks. The correlation of data obtained from SEM and DIC observations confirmed that, in defective specimens, cracks predominantly initiated from the notched area, with the fatigue crack initiation phase accounting for approximately 50% of the fatigue life. Furthermore, in edge-notched specimens, the final failure resulted from the coalescence of notch-emanating cracks.

Although the conducted experiments revealed useful trends in the performance of *YZ*-printed PLA, further research involving more extensive testing and examination of environmental conditions is required to extend the applicability of these findings.

## Figures and Tables

**Table 1:** Type I tensile specimen dimensions according to the ASTM D638 standards.

Dimensions	Value (mm)
W—Width of narrow section	13
L—Length of narrow section	57
WO—Width overall	19
LO—Length overall	175
G—Gage length	50
D—Distance between grips	115
T—Thickness	3.2
R—Radius of fillet	76
Rn—Radius of the edge notch	2
Rc—Radius of the circular hole	2

**Table 2:** Nominal and measured dimensions of specimens.

Dimensions	Nominal (mm)	Measured Range (mm)	Notes
Thickness	3.2	3.1–3.25	Deviation ≤ 0.1 mm
Width	13	12.9–13	Deviation ≤ 0.1 mm
Notch diameter	4	4	12.23

**Table 3:** Stress levels and fatigue loading conditions for smooth, edge-notched, and holed specimens.

Name	σ_UTS_ (MPa)	σ_max_/σ_UTS_	Maximum Load (N)	Minimum Load (N)	σ_max_ (MPa)	σ_min_ (MPa)	σ_mean_ (MPa)	σ_a_ (MPa)
Smooth specimen	37.23	0.6	893	45	22.34	1.12	11.73	10.61
	37.23	0.70	1042	52	26.06	1.30	13.68	12.38
	37.23	0.75	1117	56	27.92	1.40	14.66	13.26
	37.23	0.8	1191	60	29.78	1.49	15.64	14.15
Edge-Notched specimen *	36.69	0.6	614	31	22.01	1.10	11.56	10.46
	36.69	0.75	768	38	27.52	1.38	14.45	13.07
	36.69	0.80	819	41	29.35	1.47	15.41	13.94
	36.69	0.85	870	44	31.19	1.56	16.37	14.81
Holed specimen *	34.32	0.75	704	35	25.23	1.26	13.24	11.98
	34.32	0.80	751	38	26.91	1.35	14.13	12.78
	34.32	0.85	798	40	28.59	1.43	15.01	13.58

* For specimens with discontinuities, the nominal stress was determined based on the net area remaining after the defect was introduced.

**Table 4:** Mechanical properties of *YZ* and *XY* specimens from tensile testing.

Specimen Type	Num.	Young’s Modulus (GPa)	Ultimate Stress (MPa)	Elongation at Break (%)
*YZ* specimens	1	2.558	37.134	4.926
	2	2.704	37.209	4.846
	3	2.875	37.359	5.037
	Mean	2.712	37.234	4.937
	SD	0.159	0.115	0.096
	CI (95.0%)	2.318–3.106	36.949–37.519	4.699–5.175
*XY* specimens	1	2.659	31.483	6.232
	2	2.609	31.033	5.612
	3	2.716	31.168	5.881
	Mean	2.661	31.228	5.909
	SD	0.054	0.231	0.311
	CI (95.0%)	2.528–2.794	30.654–31.802	5.137–6.681
*p*-value		0.651	0.00003	0.00217

**Table 5:** Mechanical properties of edge-notched and holed specimens from tensile testing.

Specimen Type	Num.	Young’s Modulus (GPa)	Ultimate Stress (MPa)	Elongation at Break (%)
Edge-Notched *YZ* specimens	1	2.869	36.330	1.765
	2	2.843	37.050	1.825
	Mean	2.856	36.690	1.795
Holed *YZ* specimens	1	3.044	34.350	1.540
	2	2.990	34.280	1.490
	Mean	3.017	34.315	1.515

**Table 6:** Fatigue test results for smooth, edge-notched, and holed specimens.

Specimen Type	Stress Level (%)	σ_amp_ (MPa)	N_f1_ (Cycle)	N_f2_ (Cycle)
Smooth Specimens	0.6	10.611	99,940	106,880
	0.7	12.379	38,691	43,596
	0.75	13.263	24,400	24,080
	0.8	14.147	17,700	18,771
Edge-Notched Specimens	0.6	10.457	55,250	58,920
	0.75	13.071	11,756	14,992
	0.8	13.942	9607	8576
	0.85	14.814	6101	6146
Central-Holed Specimens	0.75	11.982	21,670	17,164
	0.8	12.781	12,759	9553
	0.85	13.580	10,936	9112

**Table 7:** Estimated fatigue coefficients (A and B) for each specimen type.

Specimen Type	A	B	R^2^ Value
Smooth Specimens	68.159	−0.161	0.991
Edge-Notched Specimens	57.147	−0.155	0.994
Central-Holed Specimens	48.436	−0.141	0.736

## Data Availability

The original contributions presented in this study are included in the article. Further inquiries can be directed to the corresponding author.
